# Application of the performance of machine learning techniques as support in the prediction of school dropout

**DOI:** 10.1038/s41598-024-53576-1

**Published:** 2024-02-17

**Authors:** Auria Lucia Jiménez-Gutiérrez, Cinthya Ivonne Mota-Hernández, Efrén Mezura-Montes, Rafael Alvarado-Corona

**Affiliations:** 1 Centro Universitario de los Lagos, Universidad de Guadalajara, Enrique Díaz de León 1144, Paseos de la Montaña, 47460 Lagos de Moreno, Jalisco Mexico; 2 Universidad del Valle de México, Calz. de Tlalpan 3016 y 3058 Ex Hacienda Coapa, 04910 Alcaldía Coyoacán, CDMX Mexico; 3 Instituto de Investigaciones en Inteligencia Artificial, Universidad Veracruzana, Calle Paseo No. 112, Lote 2 Col, Nueva Xalapa, 91097 Xalapa, Veracruz Mexico; 4 Centro de Estudios Tecnológicos Industrial y de Servicios N°06 “Ignacio Manuel Altamirano” , Cuitláhuac No. 50 Esq, Av. Tlahuac, Los Reyes Culhuacan, 09840 Iztapalapa, Ciudad de México Mexico

**Keywords:** Engineering, Mathematics and computing

## Abstract

This article presents a study, intending to design a model with 90% reliability, which helps in the prediction of school dropouts in higher and secondary education institutions, implementing machine learning techniques. The collection of information was carried out with open data from the 2015 Intercensal Survey and the 2010 and 2020 Population and Housing censuses carried out by the National Institute of Statistics and Geography, which contain information about the inhabitants and homes. in the 32 federal entities of Mexico. The data were homologated and twenty variables were selected, based on the correlation. After cleaning the data, there was a sample of 1,080,782 records in total. Supervised learning was used to create the model, automating data processing with training and testing, applying the following techniques, Artificial Neural Networks, Support Vector Machines, Linear Ridge and Lasso Regression, Bayesian Optimization, Random Forest, the first two with a reliability greater than 99% and the last with 91%.

## Introduction

School dropout data were considered because it has been studied from different approaches and whose figures indicate that there is work to be done^1^. Dropping out of school can be defined as the "premature abandonment of a study program before achieving the title or degree, and considers a time long enough to rule out the possibility of the student re-entering"^2^. This problem can be seen from different perspectives: psychological, economic, sociological, organizational, and interactions^3^.

Likewise, among the educational problems that Latin America shares is school failure, making it difficult for education systems to achieve successful projects for each of the school trajectories of young people. This is, without a doubt, something that alerts us daily. At the higher level, it exceeds 57%, while at the upper secondary level, it is 33%; Both percentages speak of a high school dropout rate. The Organization for Economic Cooperation and Development (OECD), in its 2017 overview of higher education, mentions that 17% of the population aged 25 to 64 in Mexico had completed higher education in 2016, the highest proportion low among OECD countries. This is 20% below the average of 37%, but higher than in some partner countries, such as Brazil (15%), China (10%), India (11%), Indonesia (10%) and South Africa (12%). It should be noted that, although higher and upper secondary education continues with great limitations, the proportion of young adults who completed their upper secondary education studies increased from 20 to 25%, while the proportion who completed higher education increased from 17 to 22%. To continue increasing educational coverage and its diversification, it is necessary to maintain joint actions among the entities involved: State, institutions, and society^4^.

As mentioned by^5^ mentions, that vulnerable and excluded students are those who have difficulties learning what is necessary and achieving good performance. These reasons lead students to stop attending classes, fail cycles, and finally drop out. Added to the above, "even harder is the fact that this failure ends up being, in large part, something "constructed" by the school itself, based on its dynamics, judgments, prejudices, and practices." p. 34.

Therefore, it is important to develop a model that helps reduce the problem of school dropouts. The development of a model is proposed that helps predict the behavior of students in dropping out of school, from the early stages of their school career. From admission, obtain information about each student so that through the model students who are at risk of dropping out can be identified and the relevant areas can attend to and guide these needs, all to reduce dropout rates. of educational institutions.

The data used for training, validation, and testing were taken from three instruments, the 2010 Population and Housing Census (CPV), the 2020 Census, as well as the 2015 Intercensal Survey (EIC 2015) carried out by the National Institute of Statistics and Geography (INEGI), which contains information on the inhabitants and homes in the 32 states of the country and its 2,457 municipalities (Table 1). The process to collect the information was through a direct interview with a suitable informant over 18 years of age, using a printed questionnaire with questions, about population, housing, and home, on multiple topics such as birth, ethnicity, education, economic issues, services of health, technological and other relevant characteristics. When working with data from three different surveys, it was necessary to carry out a homogenization process, filtering out incomplete records, and leaving only the records of people over 14 years of age. The purification of the variables was carried out by meeting some criteria, such as reviewing that each variable was present in the three data banks used and the correlation with the academic level variable, leaving eighteen variables plus two variables generated, one of them indicating the data bank and the second indicating whether I drop out, this last variable is the one predicted by the model, Table 2 lists and describes each of the variables used.

**Table 1:** Themes of the variables.

Population	Housing
Subjects	Subjects
Total population and structure	Building characteristics
Birth registration	Size and use of space
Marital status	Cooking conditions
Health services	Tenure and access conditions
Ethnicity	Access to water
Education	Sanitary facilities and sanitation
Economic characteristics	Electricity
Unpaid work	Solid waste
Migration	Equipment
Daily mobility	Household goods and automobiles
Fertility and mortality	Information and communication technologies

**Table 2:** Selected variables.

Variable	Label	Classification
ENT	Code of the federative entity	Continue
MUN	The municipality or delegation code	Continue
SEXO	Sex	Discrete
EDAD	How old is it?	Continue
SERSALUD	When his health problems, where does he/she go for treatment?	Discrete
PERTE_INDIGENA	Now I want to ask you: Does speak any dialect or indigenous language?	Discrete
NACIONALIDAD	In which state of the Republic or in which country was born? (Country)	Continue
ASISTEN	Does currently attend school?	Discrete
ESCOLARI	What is the last year or grade that passed in school? (Grade)	Continue
NIVACAD	What is the last year or grade that passed in school? (Level)	Continue
ESCOACUM	Cumulative schooling (cumulative years passed)	Continue
SITUA_CONYUGAL	Currently	Discrete
SITUACION_TRAB	At your job last week	Discrete
COMPUTADORA	Does this have a computer in this household?	Discrete
CELULAR	Does anyone have a cell phone in this household?	Discrete
INTERNET	Does this have the internet in this household?	Discrete
INGTRHOG	Monthly income from work in the household	Continue
TAMLOC	Size of locality	Continue
CPV	Year of Population and Housing Census *	Discrete
DES	Deserted*	Discrete

The model is a description of the patterns and relationships between the data. Once a predictive model is trained, it could be used effectively for future forecasts. There are various computational learning techniques to build predictive models. The models that will be used in this work are ANN, Linear Ridge and Lasso Regression, Bayesian Optimization, Support Vector Machines, and Random Forest, to have points of comparison between different techniques. These techniques were selected because they were among the most popular and competitive to solve regression problems based on the literature review. Decision trees, random forest, ANN, and Support Vector Machines were the ones that obtained the best results^6–12^. Linear Ridge and Lasso techniques were also chosen, which use a penalty structure; and Bayesian Optimization estimates conditional probabilities; The non-linear techniques selected are the Random Forest, based on decision trees, the interpretation is simpler; The Support Vector Machine is a flexible statistical model and the Artificial Neural Networks are inspired by the way the brain operates, depending on its diverse structure, it is possible to predict new instances, and it can be processed with incomplete data^13^.

The work was developed in four iterative stages, the first data collection, the second the analysis and filtering, the third the implementation of AI and statistical techniques, and the fourth and last the analysis of the results.

## Development

They are developed following the Knowledge Discovery in Databases (KDD) methodology, data selection and pre-processing, knowledge extraction, interpretation, and evaluation.

### Data collection

The research population consisted of the inhabitants of the 32 states and their 2,457 municipalities in Mexico, in the age range of 15 years and older. Data composed of population and housing variables were reviewed. Table 1 shows the topics considered in the instruments used in the data collection by^14–16^.

The survey was conducted in May 2010, March 2015, and March 2020, respectively for each instrument. It was carried out by trained personnel, based on a printed survey conducted to appropriate informants, such as heads of household or persons 18 years of age or older residing in the dwelling. The data are available in open access on the INEGI portal, in the case of the CPV 2010 the sample was 2,903,640 dwellings and 11,938,402 persons, of the EIC 2015 was 7,853,702 dwellings and 15,880,744 persons, these two instruments available two files for each federal entity, one of population and one of housing, in the CPV 2020 was a population file and another of housing made up of 4,016,627 dwellings and 15,016,309 persons^14–16^.

### Data analysis and filtering

Based on the data collected from the Intercensal Survey 2015 and the Population and Housing Censuses 2010 and 2020, conducted by the National Institute of Statistics and Geography (INEGI), the information was reviewed and a process of correlation of variables was carried out. With the desertion target variable, variables were selected from the total. Eighteen population and housing variables were considered, as shown in Table 2. The filtering was carried out by fulfilling some criteria, and checking that each variable was present in the three data banks used.

The target or output variable is DES, and the rest of the variables were considered input variables of the model. The CPV variable of the census year is the one that will indicate the prediction of the model, with the possibility of entering current data. Each of the variables is directly proportional to a correlation percentage higher than 10%.

**Figure 1:**
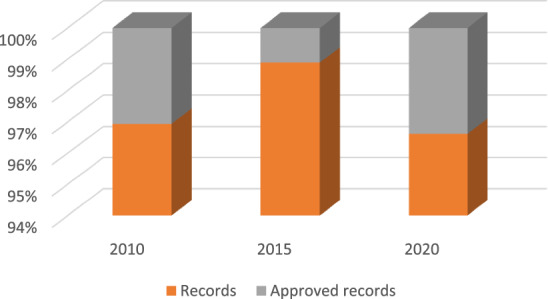
Percentage of cases obtained and standardized per instrument.

The next step, after selecting the variables, was to review the cases, discarding the records with empty values, as well as duplicate records, and the population records of persons 15 years of age and older who entered high school and higher education levels were retained. The total number of cases that were retained and homogenized was 1,080,782 from the 32 states of the country. Figure 1 shows the percentage of records contained in each database and the number that could be standardized.

Since we worked with three different instruments and data sets, it was necessary to carry out a homogenization process based on the mnemonics of each variable, its data type, size, and labels. After merging the data sets, a search was made for duplicate records, and again for records with empty data, which were eliminated. It was detected that the nationality variable in each instrument used different labels that were homogenized, leaving a value of 1 for Mexicans, 3 for foreigners, and 9 for those not identified. As with nationality, the data for the ASISTEN and NIVACAD variables were homogenized. Records containing unspecified values were discarded. Finally, we also discarded the cases of people who did not enter upper secondary and higher education. Of the cases already homogenized, 80% were used for training and the remaining 20% for testing.

The model was designed in Python with the use of the Anaconda development tool, libraries such as Sklearn, Numpy, Pandas, and Matplotlib, a tool used for computational learning and data science applications, which is a free and open distribution that works with Python and R language.

This model automates the processing of the data by performing first the training and at the end a test, with the identification of some factors and their importance in attrition. As shown in Fig. 1, we had a large amount of data, allowing us to perform complete training to identify the factors that have the greatest influence on the decision of whether a person concludes the academic level he/she is studying.

## Artificial intelligence techniques

The Artificial Intelligence techniques used were the Multilayer Regression Perceptron, the performance was compared with Bayesian Optimization, Linear Support Vector Regression Machines, and the Random Forest, better known as Random Forest. The aforementioned techniques were selected because they have been used more frequently and have obtained efficient results in multiple cases^17^, below, each of them is described.

### Artificial neural networks (ANN)

In the case of ANN, a Multilayer Perceptron was used, and tests were made regarding the structure leaving the ANN with four hidden layers with two neurons each, of the optimization algorithm was tested with three, stochastic gradient descent (SGD), Stochastic gradient descent (SGD), stochastic gradient descent based (ADAM) and the quasi-Newton method (LBFGS), from which results were obtained with an error of less than 1%, resulting optimal with ADAM, due to the amount of data that were processed, this algorithm was more efficient in the processing. A loss of 2.3E-4 was reached.

### Bayesian optimization

**Figure 2:**
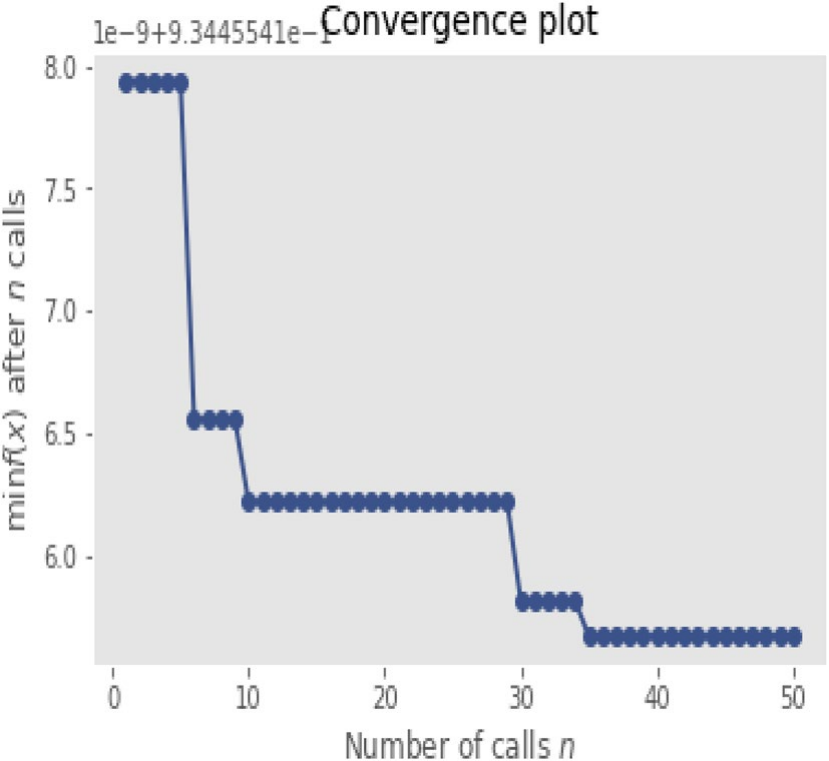
Evolution of the optimization.

A Bayesian network is a directed acyclic graph, the nodes represent each variable, and the arcs the probabilistic dependence, which from the predecessor node specifies the conditional part towards the dependent variable that points to each arc^18^. The model returns the mean of the estimator score matrix. The estimator used is the regression loss estimator of the mean square error. Figure 2 shows the evolution of the optimization over the 50 calls that were indicated in the model with Bayesian networks.

### Random forest

A decision tree is a diagram containing the parts of the name that receive a root with all the observations, the branches that are the internal nodes, and the leaves that are the ones containing the final classification^19^. A tree represents a segmentation of the data, which is created by applying a series of simple rules^20^. Each rule assigns an observation to a segment based on the value of an input. One rule is applied after another, resulting in a hierarchy of segments within segments.

The hierarchy is called a tree and each segment is called a node^21^. Thus, the internal nodes of a tree represent validations on attributes, the branches represent the outputs of the validations, and the "leaf nodes" represent the classes^22^.

The predictor DES, the predicted regression target of an input sample is calculated as the average predicted regression targets of the trees in the forest, returning the coefficient of determination of the prediction.

**Figure 3:**
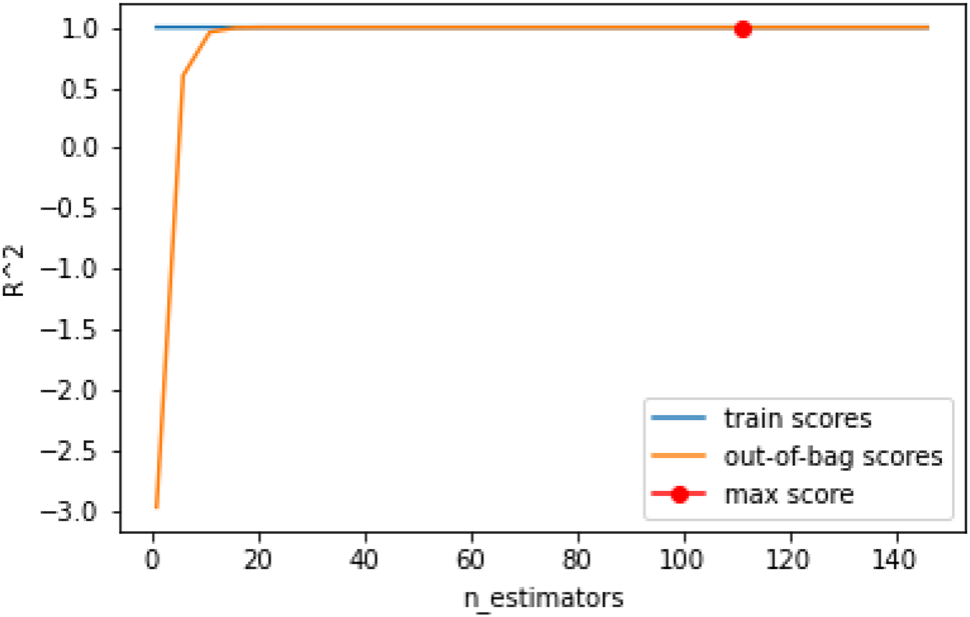
Validation using the Out-of-Bag error.

Taking the Random Forest technique which obtained competitive results we started to perform validations employing the out-of-bag error method as shown in Fig. 3.

**Figure 4:**
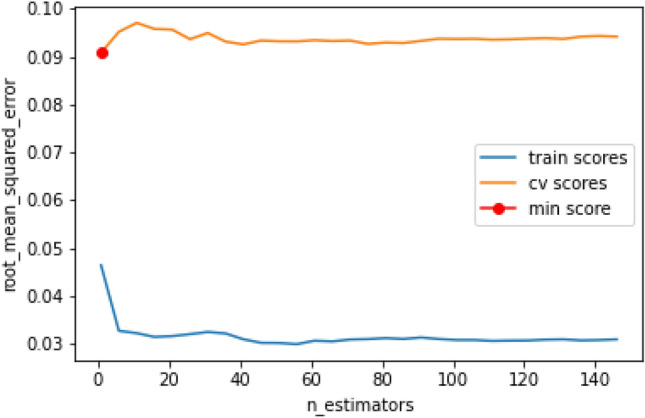
Validation using k-cross-validation and neg_root_mean_squared_error.

The validation uses k-cross-validation and neg_root_mean_squared_error is seen in Fig. 4. In the first validation, the training and output are maintained, while in the second validation, it can be seen that with the training data, the levels are closer to zero while using the validation data increased by about 5 hundredths.

### Support vector machines

Unlike most learning methods the SVM utilizes the inductive bias associated with it, minimizing the structural risk. By taking the training examples that lie on the boundaries of the maximum margins of each side of the hyperplane known as support vectors. This process helps to reduce overfitting. "The convexity property required for its resolution guarantees a unique solution, in contrast to the non-uniqueness of the solution produced by an artificial neural network"^23^.

Each of the AI techniques used obtained good training performance.

## Regression statistical techniques

The statistical technique used was Ridge and Lasso’s linear regression was selected because of its frequent use, presenting efficient results in multiple cases^17^.

### Ridge and Lasso’s linear regression

Linear regression is a statistical method that attempts to model the linear relationship between a continuous or dependent variable of the predictor variables. A linear equation is used to perform the corresponding adjustment.

**Figure 5:**
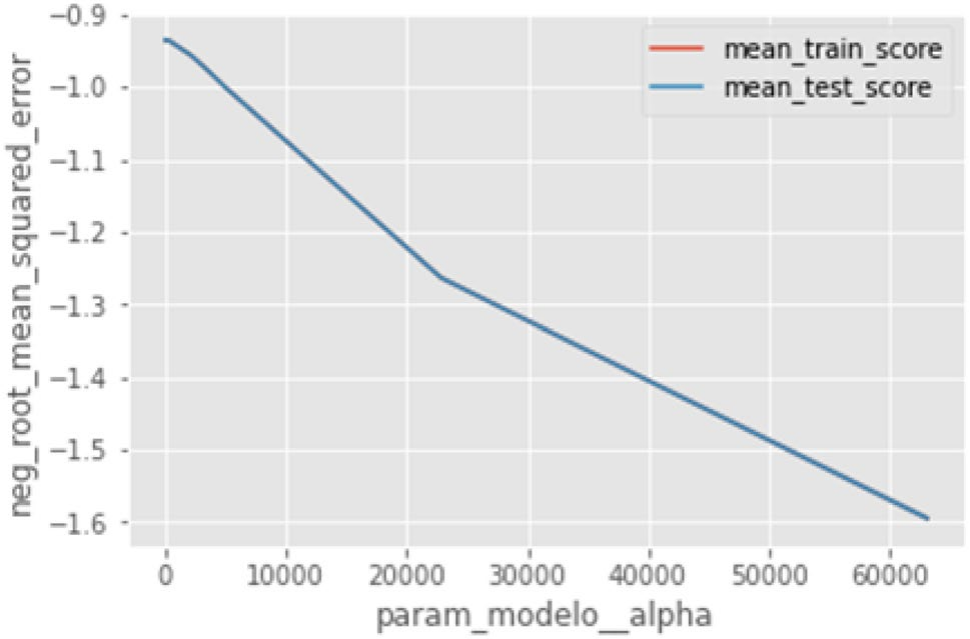
Error evolution.

In the preprocessing, the Ridge model is integrated, the hyper parameters are optimized and a search is performed with the Random Grid. The evolution of the error is shown in Fig. 5.

## Results

The results obtained from the four machine learning techniques and the statistical regression technique used and explained above were adjusted to obtain competitive results.

**Figure 6:**
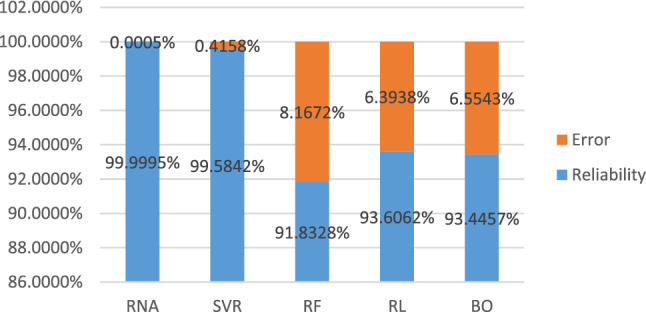
Final results of the techniques.

Acceptable reliability results were obtained in each of the implemented techniques as shown in Fig. 6 where the reliability was higher than 91%.

The results show that the techniques used had results below the percentage of error established as a convergence criterion of 10%. As for processing times, tests were carried out on three different computers with different characteristics. Table 3 shows the characteristics of each of the computers used. Table 4 shows the processing time in each piece of equipment corresponding to the implemented techniques.

**Table 3:** Computer equipment characteristics.

Equipment	1 Leo Atrox	2 Laptop	3 Desktop
Processor	36 vCPUs (Intel ® Xeon ® CPU E5-2690 v3 @2.6 GHz)	Intel(R) Core(TM) i5-1035G1 CPU @ 1.00 GHz, 1201 MHz, 4 main processors, 8 logical processors	Intel(R) Core(TM) i7-7700 CPU @ 3.60 GHz, 3600 MHz, 4 main processors, 8 logical processors
RAM Memory	150 GB	8 GB	8 GB
Storage	200 GB	500 GB	240 GB

**Table 4:** Processing times in seconds.

Technique	1 Leo Atrox	2 Laptop	3 Desktop
ANN	98.7915	93.6814	65.5440
SVR	142.008	149.3931	140.3645
RF	6277.125	**	**
RL	79.8701	445.0875	578.1248
BO	136.922	321.5258	178.071

**Results were not obtained.

The ANNs were trained in the shortest time and equipment with characteristics that most of them have access to, in the case of the random forest, for the established data, it was observed that in the equipment used only the supercomputer obtained results, the rest after starting the process were blocked and after several hours or even days, they did not conclude the training.

As the neural networks obtained the best results in the shortest time, several tests were carried out to search and compare the characteristics and attributes of the perceptron that influenced the reliability, the tests started with the number of layers and hidden neurons, and the test was complemented with the optimization algorithm and the activation function.

## Conclusions

School dropouts are a current problem in our country and around the world. In the current context, its increase as a result of the COVID-19 pandemic is evident.

To address this problem at the secondary and higher educational levels in Mexico, through the implementation of Machine Learning techniques, such as the development of ANN, results were obtained with a reliability of 99%, the Vector Machines support, Bayesian Optimization, and Random forest, and the statistical technique Linear Regression Ridge and Lasso managed to obtain results with high reliability of over 91%. The model that is decided to implement will be very useful to address desertion.

More than one million records were processed which served as training in each technique. Considering the results of reliability and processing time. The ANN was the technique that presented the highest reliability with 99%. Multiple tests were carried out with two different activation functions, varying the structure of the network, the optimization algorithm, the iterations, and the penalty parameter, highlighting the Rectified Linear Unit (relu) activation function and the ADAM optimization algorithm. Of the five models, the one that requires the most computing power to process is Random Forest.

Each of the Machine Learning techniques implemented, allows us to identify the probability that a young person may be at risk of dropping out, for reasons beyond his control, who is in a vulnerable situation in continuing his studies at the upper secondary level or higher, and what federal and educational institutions can provide support for its improvement.

It is worth developing models that allow improving social problems such as school dropouts, applying them and trying to direct each social program of educational institutions and the government; as well as joining forces with the objective that people who need it can reduce the gaps and equalize opportunities for study and professional growth for the entire society, which is a core task.

To complement future work, it is expected to develop an open platform that allows each institution to achieve the appropriate diagnosis to provide the support that each student requires.

## Data Availability

The data used is available in the links that are shared below, to access it you only need to open the site, and make sure that you are in the open data tab and in the option to download all the files. [Population and Housing Censuses 2020] [https://en.www.inegi.org.mx/programas/ccpv/2020/#open_data]. [Population and Housing Censuses 2010] [https://en.www.inegi.org.mx/programas/ccpv/2010/#open_data]. [Intercensal Survey 2015] [https://en.www.inegi.org.mx/programas/intercensal/2015/#open_data].
